# Expertise-Related Differences in Cyclic Motion Patterns in Drummers: A Kinematic Analysis

**DOI:** 10.3389/fpsyg.2020.538958

**Published:** 2020-11-10

**Authors:** Eckart Altenmüller, Wolfgang Trappe, Hans-Christian Jabusch

**Affiliations:** ^1^Institute of Music Physiology and Musicians’ Medicine, Hanover University of Music, Drama and Media, Hanover, Germany; ^2^Institute of Musicians’ Medicine, University of Music Carl Maria von Weber Dresden, Dresden, Germany

**Keywords:** drumming movements, cyclic motor patterns, musical expertise, motion capture, motor learning

## Abstract

**Background:**

At present only little information is available concerning the acquisition of skilled movements in musicians. Although optimally a longitudinal study of changing movement patterns during the process of increasing expertise is required, long-term follow up over several years is difficult to manage. Therefore, in the present cross-sectional study a comparative kinematic analysis of skilled movements in drummers with different levels of expertise was carried out.

**Aims:**

The aim of the investigation was (1) to analyze the kinematic differences between beginners, students and expert drummers, and (2) to deduce from the results general rules related to the acquisition of drumming expertise and (3) to discuss the implications for drum teaching.

**Method:**

Two highly skilled experts, eight professional drumming students and five beginners participated in the experiment. Fast repetitive drumming movements were assessed using an active infrared measurement setup (SELSPOT-System). Recording was obtained from LEDs positioned over the shoulder-, elbow-, wrist- and MCP-joints and close to the tip of the stick at a sampling rate of 300 Hz. Kinematic analysis included calculation of angles, velocities and accelerations and assessment of the relation between velocity and acceleration as phase diagrams.

**Results:**

Temporal accuracy of the drumming movements was related to expertise. In contrast to beginners, experts and students revealed a high degree of self-similarity of movements and a predominant use of low-mass distal joints, resulting in a whiplash-like movement when hitting the pad.

**Conclusion:**

Intense training in students and experts results in economic utilization of forces. Percussion teachers can take advantage of the kinematic analysis and improve their instructions according to the student’s observed motor pattern.

## Introduction

The precise execution of very fast and in many instances extremely complex movement patterns characterizes the motor skills of professional musicians. To gain such a high level of expertise prolonged and intense training over many years is a prerequisite. A classical pianist for example has practiced his instrument more than 10,000 h before entering the “professional” level and performing in public (Ericsson et al., 1993). Basis of a musician’s career is “technique,” which comprises excellent control of movements in both, temporal and spatial dimensions, high velocity of these movements and the ability to sustain regularity, speed and loudness of playing over a long time period. Consequently, these “technical” training goals are implicitly defined in all major collection of “Etudes,” often composed by outstanding performer-composers, e.g., Chopin, Skriabin, Rachmaninov, Prokofiew to name but a few for the piano. And it is not by chance that a Russian interpret and piano teacher, Neuhaus (1967, p 72), has formulated in his pioneering book on the art of playing piano: “The most difficult thing in playing the piano (with respect to the physical process) is to play very long, very fast and very loud.” With respect to the biomechanics playing an instrument requires a maximized economy of force at a minimized error rate.

Although the acquisition of professional skills in musicians represents an excellent paradigm to study the neurobiological and biomechanical foundations of sensory-motor learning in general, only in the last 25 years high quality studies using Motion Capture were devoted explicitly to these special abilities. Engel et al. (1997) used three-dimensional Motion Capture (MoCap) in combination with MIDI-based performance analysis and recorded the movements of the right wrist and fingers of pianists. They were playing scales with identical beginnings and different continuations, either including or excluding thumb-under movements. The aim of the study was to ascertain in which way hand and finger kinematics diverged prior to the depression of the last common note. As a result, playing an ascending scale with the requirement of a thumb-under maneuver could evoke an anticipatory modification as much as 500 ms in advance of the last common note as compared to scale playing without thumb-under movement (Engel et al., 1997). In view of the findings, it was suggested that a strict serial execution of a movement sequence was favored as long as this was compatible with the demands of the tasks. Sforza et al. (2003) investigated the repeatability of finger movements during scale playing in different pianists with different experience using MoCap. Repeatability of movements of different recordings was analyzed for each finger by superimposing the traces of the vertical coordinate of the fingertip of the different recordings as a function of time. Comparison of the areas under these superimposed graphs revealed repeatability between 65.8 and 81.4% (mean superimposition) in five different pianists. Repeatability was found lower in experienced pianists than in learners and teachers. As a possible explanation, the experts were suggested to be more focused on other aspects of musical performance than on hand and finger movements. This might be in contrast to less experienced pianists who might be more focused on the movements required for execution of a basic task such as scale playing (Sforza et al., 2003). Palmer and Bella (2004) used a combination of MIDI and MoCap analysis using video cameras and passive markers on hands and fingers of pianists for investigation of the relation between tempo and movements. Pianists repeatedly performed short melodies with increasing tempi until they made errors. Most tempo-related changes in the variability of finger movements were observed in the in the plane perpendicular to the keyboard. Peak amplitudes of motion before striking the keys increased as tempo increased. It was concluded that increased movement amplitudes at faster rates may reduce or compensate for speed/accuracy trade-offs. In a continuing study, Goebl and Palmer (2013) investigated very thoroughly professional pianists and noted during the execution of very fast sequential five-finger-movements in keeping with the results of Sforza high individual variability, however, also clear hints for more efficient motor patterns in the best pianists, with predominant use of lumbrical muscles and optimization of the biomechanical parameters, such as finger force and impact when hitting the keys.

Focusing on changes in motor patterns with increasing expertise, Furuya and Kinoshita (2008) used a 2D motion capture system and employed an octave-striking task to compare repeated strokes of experts and novices; they found more consistent and efficient movements in experts than in novices. Using another task (a tremolo spanning the interval of a sixth) and 3D motion capture, they found similar results: Professionals tended to perform with larger degrees of freedom, used less muscular force, and generated the motion from more proximal parts (wrist rotation) of the movement chain than did novices. In a further study, Furuya et al. (2011) investigated interjoint coordination in pianists through upper limb kinematics and muscular activity patterns. Expert pianists were shown to have smaller movement range from distal joints and greater muscular activity from extrinsic upper limb muscles when compared against novice pianists. With increasing tempo, expert pianists were found to consistently make use of proximal rather than distal joints.

Although pianists were preferred subjects in MoCap studies, mostly due to the visibility of finger- and hand movements with camera systems and to the ease of recording the playing using MIDI-technology MoCap has been used in the last years also in other instrumentalists, for examples in string players (Shan et al., 2007; Schoonderwaldt and Altenmüller, 2014; Ancillao et al., 2017; Hopper et al., 2017) and wood-wind players (e.g., Albrecht et al., 2014) yielding interesting results in terms of precision of timing of movements. In the last years increasing interest emerged in measurements of musician’s movements during ensemble synchronization (e.g., Demos et al., 2017, for a review see Keller et al., 2014) and in and emotional gesturing of musicians (e.g., Masanobu et al., 2012). Since these measurements did not attempt to assess changes related to increasing expertise, we do not further review this work here.

Dahl (2004) was the first to publish a larger study on the above-mentioned movement analysis techniques to individual drummers. She was able to demonstrate flexible wrist movements, moving the stick in a “whiplash” motion. Skilled players appeared to maintain their movement strategy consistently for different playing conditions, but movement trajectories differed considerably in between different individuals. The dynamic level influenced both the height to which the stick was lifted in preparation for a stroke, and also the timing of strokes. Intervals beginning with accented strokes tended to be prolonged more at soft than at loud dynamic levels. These results were deepened in a further study using refined acoustical analysis of resulting drumming sounds (Dahl and Altenmüller, 2008) and also utilized in comparing healthy drummers with drummers suffering from dystonia (Dahl et al., 2011). Here MoCap was able to objectively monitor the co-contraction of wrist flexor and extensor muscles in patients suffering from drummers’ dystonia. Taking this approach further, Dahl and colleagues focused on expertise dependent movement fluency in different instrumentalists (Gonzalez-Sanchez et al., 2019). Starting from coarticulation, defined as the integration and fusion of otherwise separate and distinct sequential movement elements into single units, increasing expertise leads to refinement of these movement patterns (Godøy et al., 2017). According to Gonzalez-Sanchez et al. (2019), the amount of coarticulation is largely governed by the duration and rate of movement events. Additionally, the fusion of events in coarticulation is determined by anticipation and preparing of subsequent movement events. For instance, violin players have been shown to adjust their fingering depending on whether a finger controls note onsets or pitch (Baader et al., 2005), and drummers can start the upward wrist movement in preparation for a louder stroke already during the preceding stroke (Dahl, 2004).

Summarizing the reported studies on movement analysis in musicians in a more global way, three basic principles emerge:

(1) Precision of timing and dynamics of movements depends primarily on auditory feedback: “What musicians do not hear, they do not program.”
(2) Movement patterns are executed in a serial and parallel manner. Parallel execution of movements comprising multiple joints and fingers is a prerequisite for anticipatory movements. Anticipatory movements in turn (for example prior and during the thumb passage in scale playing) are a characteristic feature of expert musicianship.
(3) Movement strategies in expert musicians vary inter-individually, depending on biomechanical properties of the movement apparatus, on the physical properties of the musical instrument and on the individual internal representation of the intended sound quality, the “Sound-Ideal” (German “Klangideal”).

Up to now, only limited information is available concerning the acquisition of skilled movements in drummers. Although optimally a longitudinal study of changing movement patterns during the process of increasing expertise is required, long-term follow up over several years is difficult to manage. Therefore, in the present cross-sectional study a comparative kinematic analysis of skilled movements in drummers with different levels of expertise was carried out. The first aim of the investigation was to analyze the kinematic differences between beginners, students and expert drummers. The second aim was to clarify, whether we could deduce from the results general rules related to the acquisition of drumming expertise and draw conclusion with respect to teaching percussion.

## Materials and Methods

The specific aim of the study was to delineate parameters characterizing the movements of drummers as a function of their levels of professional expertise. Therefore, we compared healthy male drummers with different levels of expertise, two internationally renowned percussionists, eight students of percussion of the Hanover University of Music, Drama and Media and five non-percussionists. All subjects gave informed consent according to the declaration of Helsinki. The local ethics committee of the Leibniz University Hannover approved the study protocol (LUH EK2309). Subjects were right-handed according to the Edinburgh Handedness Inventory (Oldfield, 1969). Age and cumulative life practice time as an indicator of expertise are displayed in Table 1. for all individuals.

**TABLE 1:** Age and cumulative life practice hours in the subjects.

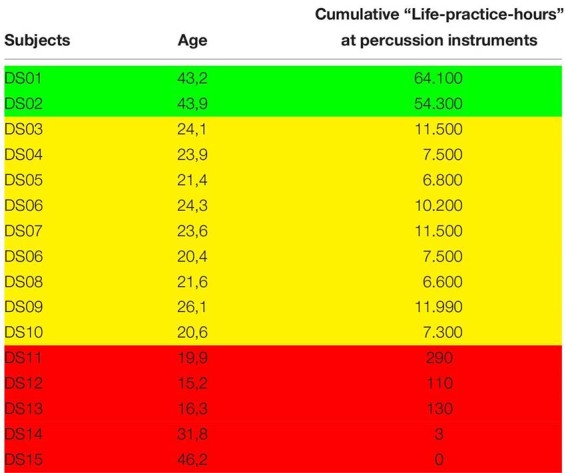

*All subjects were male and right-handed. Green, experts; yellow, professional students; red, beginners.*

**FIGURE 1:**
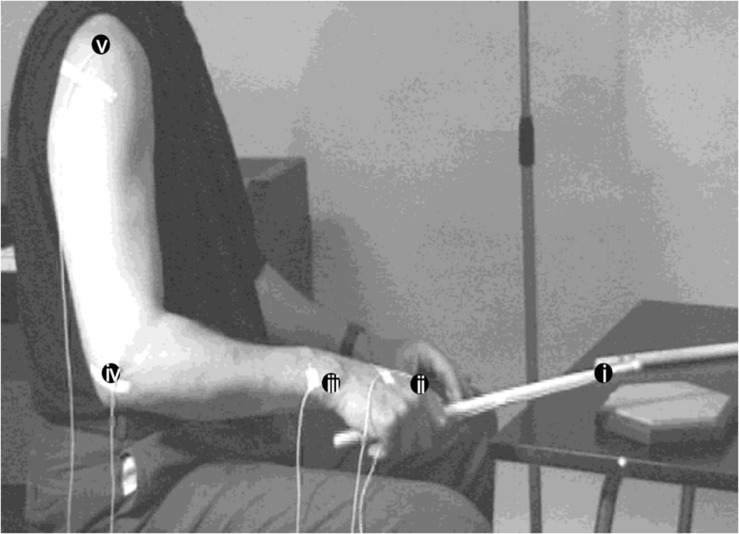
Experimental setup. Expert DS01 with LEDs attached to the stick and to lateral bony parts of right arm joints: **(i)** stick, LED is attached 3 cm below the tip of the stick, **(ii)** metacarpophalangeal joint (MCP) of the index finger, **(iii)** ulnar tuberositas of the wrist, **(iv)** epicondylus lateralis humeri of the elbow, and **(v)** tuberculum majus of the shoulder.

Subjects had to perform two different drumming exercises with their right hand on a drum practice pad (EVANS RF-12 G Practice Pad^R^). This specific drum pad was chosen, since it has excellent rebound qualities, indistinguishable from a real snare drum. The exercises were to play (a) repetitive quarter notes in forte and (b) repetitive sixteenth notes in forte at a metronome-paced tempo of 76 beats per minute (bpm) for a quarter note. Subjects were instructed to play as regular as possible and stay with the metronome and keep an even loudness. Each exercise had to be performed for 20 s. Drumming movements were recorded with an active three-dimensional movement analysis system (Selspot, Selcom Laser Measurements, Sweden) using five infrared-light emitting diodes (LEDs) attached close to the tip of the drumstick (i), and to four bony marks. Figure 1 shows the experimental setup and the position of the attached LEDs. Examples of the respective drumming tasks can be viewed in the Supplementary Material Videos 1 and 2.

Four infrared sensor cameras from different viewing positions acquired the signals of the five LEDs. Data were recorded at a sampling rate of 300 Hz and transferred to a computer for calculation of 3D-coordinates. Analyses of movement kinematics comprised calculation of solid angles, velocities, and accelerations. Besides these basic analyses, the relation between acceleration and velocity was calculated and displayed graphically as phase-diagrams. As a prerequisite, data had been smoothed using a 5th and 9th order Savitzky-Golay smoothing algorithm.

## Results

**FIGURE 2:**
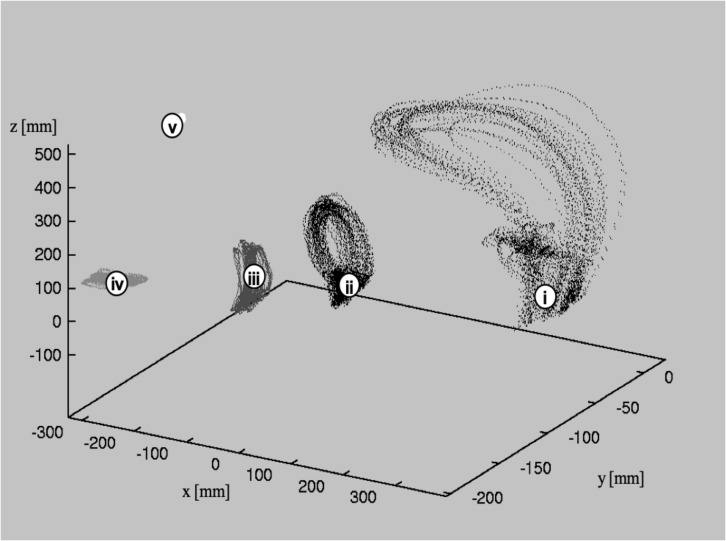
Motion trajectories of LEDs seen from the same perspective as in Figure 1 in the same skilled expert during playing quarters at 76 bpm in forte. Around 25 beats are accumulated over time. Labels of LEDs are analog to those in Figure 1. For details see text.

In Figure 2, a typical example of the drumming trajectories in an expert (DS1) playing quarters at 76 bpm forte is shown. About 25 beats are accumulated over time, each LED position yields a cyclic pattern.

Movements of the shoulder are minimal and can be seen as a spot-like trajectory in the left upper part of the graph. The largest movements occur in the stick and the wrist trajectories. The relative uniform trajectories over time demonstrate the self-similarity of movements typical for highly automated regular motion tasks. As a special drumming technique, DS1 uses “in-between”-beats at this slow speed to improve temporal accuracy of drumming. This can clearly be recognized as an additional slope in the stick trajectory.

### Accuracy in Time

**FIGURE 3:**
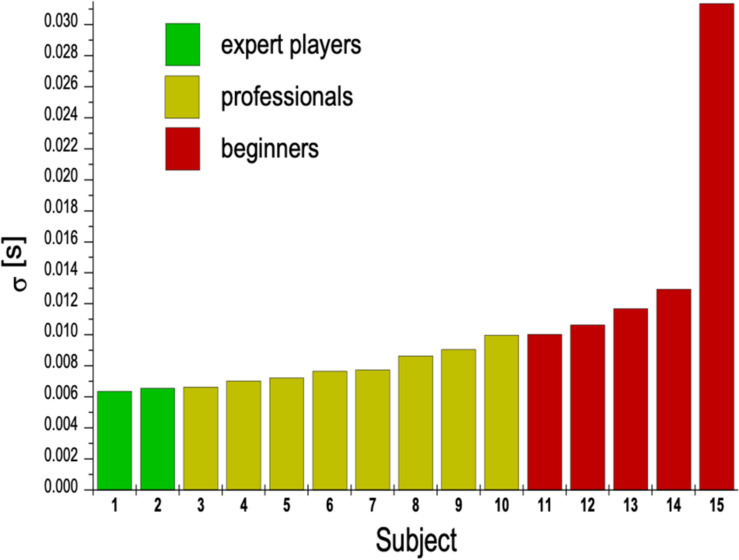
Diagram of mean temporal deviation σ[s] in time intervals between succeeding strokes while playing at a fast speed (16th notes at 76 bpm, forte). Temporal accuracy is highest in the two expert performers, less in the students, and markedly reduced in the five non-drummers.

As a measure of accuracy in time, the mean time intervals in between 60 strokes during exercise (b) and the standard deviations were calculated. Figure 3 shows the temporal deviation from the mean time interval between the strokes in each individual subject. Since subjects were asked to play at the defined speed of 76 bpm, the “optimal” time interval was 198 ms between two strokes. Low temporal deviation is equivalent to high accuracy in the time domain during playing at this fast speed. As can be seen in Figure 3, deviations from the “target”-interval ranged between 6 and 31 ms.

The order of accuracy roughly corresponds to the level of professional expertise. The differences between the two experts and the students are negligible, but between non-drummers and students they are considerable. This is in line with the general notion, that the first improvements take place after a relatively short time-period of exercise, but to *perfect* the performance, musicians have to spend many years of practice in order to achieve outstanding temporal accuracy (Ericcson, 1997).

### Trajectories and Angles

**FIGURE 4:**
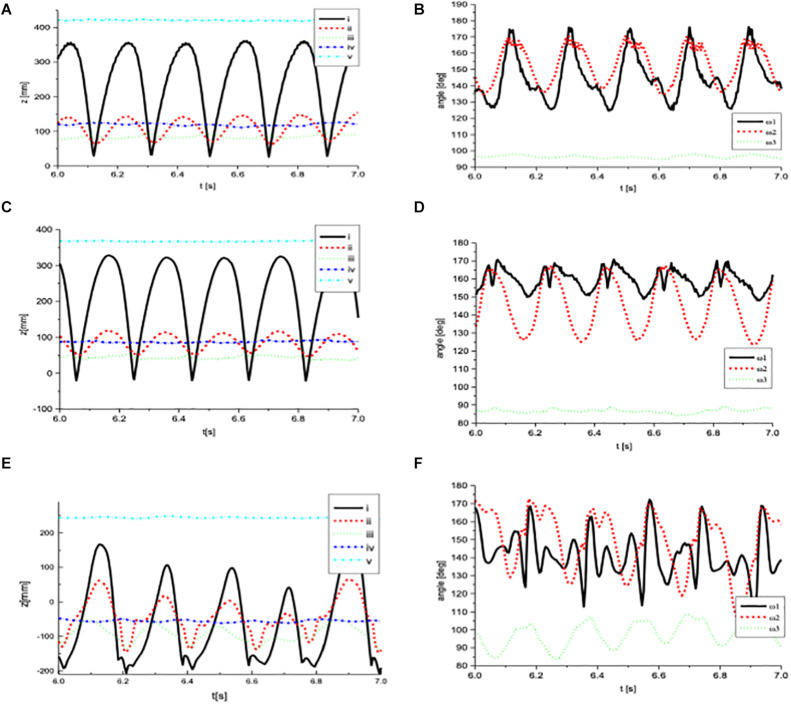
Figures on the left side show the movements of the LED positions i–v along the (vertical) z-axis over time during exercise b of **(A)** an expert percussionist, **(C)** a student, **(E)** a non-percussionist. Figures on the right side **(B,D,F)** are displaying the respective calculated angles ω_1_ between the stick and the hand at the MCP joint of the index finger, ω_2_ at the wrist and ω_3_ at the elbow (same order top – down as trajectories).

Trajectories and angles are appropriate means to characterize individual motion patterns. In Figure 4, kinematic data of three representative subjects of the respective groups (experts, students, and non-drummers) are shown. The projection of the LED positions on the (vertical) *z*-axis (see Figure 2 for visualization of the three axes) is displayed. Note that the calibration plane xy is not at the same height in different subjects, depending on the respective individual body size. The *z*-axis was chosen because the main components of movements occur in this plane.

In the left side of Figure 4, movement data over time are displayed for an expert (DS01, Figure 4A), a student (DS03, Figure 4C), and a non-drummer (DS14, Figure 4E). On the right side of the figure, dynamic changes of angles over the same time period, calculated from the original three-dimensional data are displayed over time. In this graph, the impact on the pad occurs when the angle ω_1_ and ω_2_ are at about 180 degrees (top of the line-drawing). The 180-degree angles reflect the relative extension of the wrist following the previous dorsal flexion. The elbow joint remains at a fairly constant angle of 90 degrees during the complete cycle.

As can be seen in Figure 4A, the movements of the expert are characterized by regularity and high temporal and spatial accuracy, indicating a high degree of motor control. Furthermore, the data demonstrate that the expert predominantly uses the wrist and employs a lever-like movement between the stick and the hand with the fulcrum between the thumb and the index finger (close to the MCP joint II, by approximation). In the trajectory traces, the most prominent excursions can be detected in the stick (solid black line), at the MCP joint (dotted green line), and in the wrist (dashed red line). The elbow and the shoulder trajectory demonstrate the relatively stationary position of these LEDs during the fast and forceful drumming. In the angle diagram (Figure 4B right), the prevailing use of the wrist and the employment of the lever-like movement between the stick and the hand can be detected more clearly, resulting in high angular excursions in ω_1_ and ω_2_. The contribution of the elbow joint is minimal. In consequence, a flexible, whip-lash-like motion pattern can be observed, allowing high accelerations at the tip of the stick. It should be noted that in the angle-diagram ω_1_ no or only very small reversed peaks can be detected. The accurate control of the muscular interplay in this expert results in an anticipatory lifting of the wrist immediately prior to the impact, avoiding the reaction impulse of the impact which is reflected in reversed peaks (see Figure 4B black line). The relations between the different angles demonstrate the predominant use of the distal parts of the lower arm and wrist involving smaller masses of forearm, wrist and hand.

In the professional student (Figures 4C,D), the employment of the lever-like movement between the stick and the hand is not as pronounced as in the expert. The excursions at the MCP joint II (ω_1_) are reduced and reversed peaks can be observed during every impact, demonstrating a certain lack of muscular coordination.

Compared to the expert and the student, the beginner DS14 (Figures 4E,F) demonstrates a different movement pattern. Lack of precision and non-existing self-similarity of cycles is obvious. For example, the irregularity in the trajectories of the stick and the MCP joint hint at a low degree of automatization and evidently lead to an impairment of the evenness of sound production. Moreover, in contrast to the expert and the student, pronounced movements can be observed in the elbow joint (angle ω_3_). This is due to the fact, that the subject is unable to control the movement appropriately with the wrist and between the hand and the stick. As a consequence, a stiffening as well as irregular movements of the wrist and at the MCP joint occur, which in turn leads to the necessity of predominant utilization of the elbow joint to produce the stroke movements. It should be noted that in Figure 4F the stiffness of the wrist joint is masked by the pronounced reversed peaks and backstrokes of the stick with a very irregular trace in the ω_1_. Although it is difficult to see in the angle diagrams, the particular traces of ω_1_ and ω_2_ are due to the predominant reaction of the stick and the wrist joint subsequently to the impact on the pad. Since control of the stick is poor, every impact leads to repulsion toward the palm of the hand. The reaction on the impact is also documented in the knotting of the traces of angle ω_2_ in synchrony with the angle ω_1_. In summary, the subject has not learned to adjust his muscular coordination to the physical properties of the integrated body-instrument system since the movement is disturbed by every pad stroke.

### Velocity and Acceleration

**FIGURE 5:**
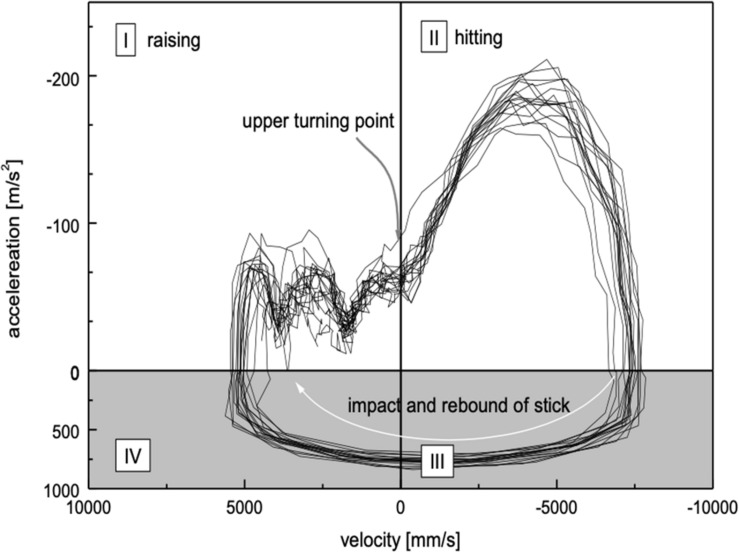
Phase plot of kinematic data of the LED attached to the stick in expert DS01 during 25 cycles of exercise b. Plotted are acceleration (*y*-axis, mm/s^2^) versus velocity (*x*-axis, mm/s) of LED movements along the (vertical) *z*-axis. The orientation of the motion is shown with the arrow (clockwise). In quadrant I the upward motion after the impact is shown. The (positive) velocity of the stick decreases until the upper reversal of the movement at velocity “0” occurs. Quadrant II shows the phase of acceleration toward the drum pad, when the subject applies movement energy to the stick for the generation of the sound on the drum. In the gray area (quadrants III and IV), the velocity changes from negative to positive values in the moment of the impact. This is an extremely short period with strong “negative” deceleration (note the different scales on the *y*-axis).

In a further step of analysis, velocity and acceleration were calculated from the motion data. A better understanding of the data and additional insights are provided by graphs depicting velocity versus the acceleration. In Figure 5, velocity versus acceleration of the LED attached to the stick is shown in the same expert as in Figure 4 for LED movements along the (vertical) *z*-axis. An animated point would go clockwise on the line through quadrants I, II, III, and IV. About 15 movement cycles are superimposed. Important for the understanding of the movement pattern are quadrant I, representing the movement away from the pad and quadrant II, representing the movement toward the pad. Quadrants III and IV (gray area) represent the very short phase of the impact on the pad. Velocity can attain positive or negative values. Positive values denominate movements toward the pad, negative values movements away from the pad. The acceleration, too, can attain positive or negative values. Negative values represent acceleration toward the pad; positive values the deceleration after the sudden impact on the pad.

The low degree of scattering of the traces shows the accuracy and the self-similarity of motion cycles in this expert. Furthermore, a constant acceleration in quadrant I (during the upward movement) can be observed, which is due to gravitational forces and intrinsic damping of the biomechanical properties of the muscles. This implies that kinetic energy is transformed into potential – anti-gravitational – energy, and only a very small amount of energy has to be applied actively by muscular contraction. In other words, a highly economical use of force – and therefore of energy – can be deduced from these traces. In quadrant II, during the downward movement toward the pad, the potential energy is efficiently transformed into kinetic energy. But furthermore, muscular force is applied, which is reflected in the increasing acceleration resulting in high velocity of the stick.

**FIGURE 6:**
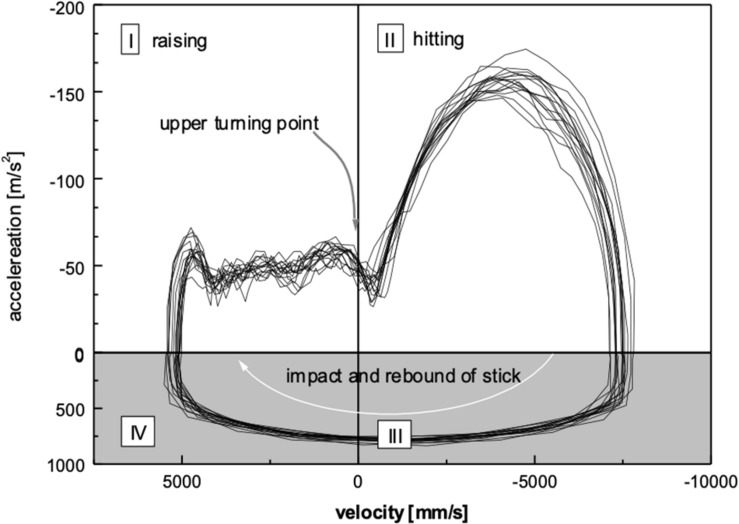
Phase plot for the student UK. Same conventions as in Figure 5. Compared to the expert, the phase-plot diagram is very similar, although minor deviations can be observed in the quadrant 1, probably due to individual biomechanical properties.

The velocity-acceleration diagram of the student (Figure 6) resembles in its characteristics the expert’s diagram. It is noteworthy that the fine structures of traces during the constant acceleration in phase I have many similarities in both, the expert and the student, revealing a triphasic pattern and a sudden onset of increasing acceleration in phase II after reaching the upper turning point.

**FIGURE 7:**
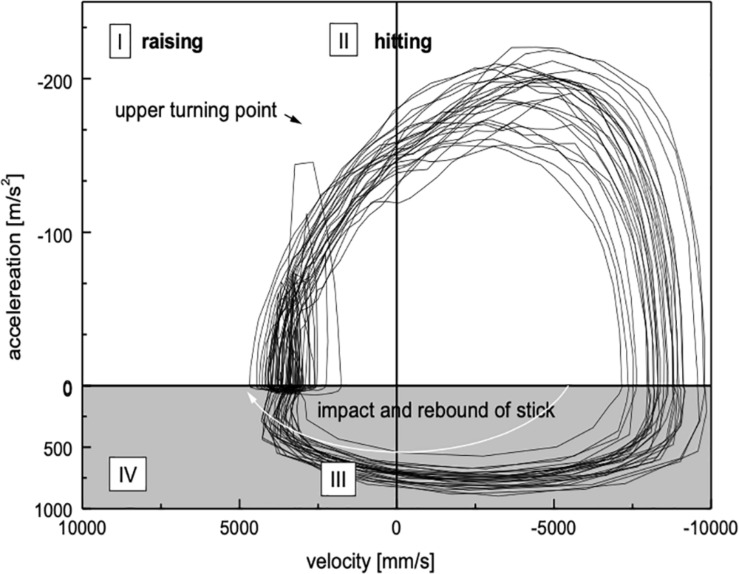
Phase plot for the non-drummer DP. Clear differences emerge compared to the traces in Figures 5, 4. The “artifacts” in quadrant I are due to a lack of co-ordination of the impact. Furthermore, the increasing slope already during the raising of the stick demonstrates an uneconomical use of force.

The non-drummer’s diagram (Figure 7) finally show some marked differences to the experts. First, due to the relatively high variability of movements, the traces are considerably scattered. Secondly, during upward movements (quadrant I), acceleration is dramatically increased. This reflects an unusual early active contraction of muscles participating in the drumming movement, which does not take place in students and experts. Such a lack of a “passive” relaxation during the upward movements can be interpreted as an uneconomical use of forces.

## Discussion

The results of the present study can be summarized as follows:

Instrumental expertise is reflected in

(a) accuracy of both, timing and amplitude of movements,
(b) predominant use of distal “low mass” parts of the body and the body-instrument system, probably as an optimization strategy for economy of forces,
(c) recurrent characteristic movement patterns, determined by both, skill and biomechanical factors.

Professional musicians are trained to execute complex and prolonged movement patterns at high speed and full volume. But the most demanding features of these movements, surpassing the level of more or less pure athleticism, emerge from the requirements concerning their quality. Making music is the only complex sensory-motor action of the upper extremities, which relies on the auditory pathway as a feedback system. The musician’s – and in many instances the listener’s ear is a highly skilled pattern analyzer, revealing inaccuracies in timing in a range of a few milliseconds and inaccuracies in meeting a given target in a range of less than millimeters. A professional musician, at least in the typical frame of a classical western orchestra, is expected to reproduce movements almost perfectly with high reliability. Additionally, the execution of movements is subject to many more or less implicitly mediated traditional rules of interpretation, especially the requirements of high regularity in timing and loudness of cyclic movements such as trills or drum rolls. It is trivial to state that these specialized sensory-motor skills require extensive training periods over many years, starting in early childhood and passing through stages of increasing physical and strategic complexities.

With respect to temporal accuracy, the data presented here reflect long-term training processes. The two internationally renowned experts attained higher degrees of temporal accuracy compared to the students and the non-drummers. The control of amplitude of drumming movements was similar to those of the students. Compared to non-drummers, both other groups executed much more regular stick movements. Besides the above-mentioned temporal accuracy, another – more subtle – training effect could be observed, namely the anticipation of the wrist movement prior to the impact on the pad. According to Soechting et al. (1996) such an anticipatory modification of kinematic elements seems to be linked to movements with high temporal constraints, as it is typically the case in drumming. This has been recently confirmed, elaborated and extended by Gonzalez-Sanchez et al. (2019).

Few studies have investigated temporal accuracy in professional musicians. Wagner (1971) assessed the rhythmical precision of playing a C-major scale in professional pianists. Correspondingly to the present results he found at a required speed of about six key-strokes per second a standard deviation of 6–10 ms in a group of 11 pianists when calculating the temporal deviations of 30 subsequent keystrokes. Moore (1992) found an even higher degree of regularity of cyclic trill movements in pianists: During a 3 s period of trills, the two notes of the trill differed in their average on-, off,- and cycle durations by less than 1 ms. However, such an outstandingly high degree of precision seems to be an exception since it has not been replicated in later investigations. Jabusch et al. (2004) used MIDI-based analysis of piano performance as an indirect measure of finger motion in scale playing. In a study with thirteen professional pianists, the evenness of scale playing in the *legato* style was investigated with special focus on hand and finger coordination in the thumb-under movements in the ulnar playing direction as well as finger cross-over maneuvers in the radial playing direction. Over all scales of all pianists, the mean standard deviation of inter-onset intervals was 8.1 ms in the ulnar playing direction and 8.9 ms in the radial playing-direction indicating a high level of evenness in playing. Expertise-related temporal precision as seen in our results has been reported recently in amateur and expert drummers: a high number of life practice hours predicted an increased temporal precision in a variety of drumming tasks (Beveridge et al., 2020). Of course, temporal accuracy is dependent on auditory perception. According to Friberg and Sundberg (1995), the absolute just noticeable differences for temporal deviations in isochronous sequences of sounds was found to be approximately constant at 6 ms for tone-inter-onset intervals shorter than 240 ms, a condition which holds for the 16th tasks in our experiment.

Besides the temporal and spatial precision of movements, musicians are confronted with a second critical demand: the ability to perform complex movements over a long time period. This is especially true for percussionists, since orchestral pieces can require fast high-rate drumming at forte over more than 20 s. It is therefore mandatory to use muscles with the greatest possible economy of force. The present data demonstrate this skill in both, experts and students. Not only the predominant use of low mass distal joints but also the benefit from gravity could be observed in all trained subjects and therefore must be regarded as a general rule. The training induced “distalization” of movements has been observed according to a personal communication of our subject DS01 by many drumming pedagogues and is communicated to their students as a training goal. With respect to more general rules of motor learning, other investigators have described this phenomenon previously. Bernstein (1947, 1988) already has hinted upon the tendency to use more distal limbs when goal directed movements are automated during exercise. Whiplash-like movements have more recently been described in pianists performing fast octaves (Furuya and Kinoshita, 2008; Furuya et al., 2011) and in other instrumentalists (e.g., Gonzalez-Sanchez et al., 2019). Such a “distalization” and whiplash-like movement pattern has as a major advantage since central nervous representations of more distal parts of the upper limbs, hands and fingers are larger and receptive fields are smaller allowing for an optimal control of movements (for a review see: Altenmüller and Furuya, 2016). On the other hand, motor behavior of non-drummers with predominant use of proximal joints and stiffening of the wrist by co-contraction of wrist flexor- and extensor muscles is a characteristic feature of untrained movements with reduced the degrees of freedom as was already noted by Bernstein, 1947. In the above mentioned study on amateur and expert drummers, electromyography showed decreased wrist muscle co-contraction in drummers with higher levels of expertise (Beveridge et al., 2020).

It should be emphasized that, albeit there are some general rules, which are in common in the group of students and experts, individual characteristics are reflected in the present analysis as well. The details of the individual phase diagrams are highly reproducible but inter-individually variable, corresponding to individual biomechanical properties of the arm. As one weakness of the study, biomechanical analyses of the upper arm, EMG-, or ultrasound measurements were not taken. Furthermore sound analysis was not possible due to the restrictions to utilize a practice drum-pad. These shortcomings will be remediated in future studies, implementing the methods for acoustical analysis and EMG analysis developed in our labs (e.g., Schoonderwaldt and Altenmüller, 2014; Ioannou et al., 2016; Beveridge et al., 2020).

In summary, highly trained drummers are able to control their instrument in a subtle but precise and efficient way. Extensive training results in excellent temporal and spatial accuracy, the benefit from gravity and the distalization of movements. The tuned phases of kinetic chains from the shoulder to the stick produces a “whip-lash-like” movement, with maximal energy brought “to the point” of the pad.

What can a drumming teacher learn from such a study? To prevent overuse injury maximal economy of force is mandatory. The data presented in this paper enables the pedagogue to observe a student in a more specific way and to control his technique with respect to use of joints and gravity, either by means of conventional video-technique or even by direct visual observation. Teachers will preferably obtain such an optimized movement pattern by encouraging kinesthetic perception and awareness of unnecessary co-contractions. Here, training in slow tempo with variable movements-patterns and stepwise refinement of motor control through kinesthetic awareness and auditory control will be helpful. Furthermore, according to the OPTIMAL theory of Wulf and Lewthwaite (2016), external focus during later phases of automation and motivation of students through autonomy seem to be very beneficial. However, experimental proof of the latter predictions is still missing.

## Data Availability Statement

The datasets generated for this study are available on request to the corresponding author.

## Ethics Statement

The studies involving human participants were reviewed and approved by the Ethics Committee of the Leibniz University Hannover, LUH EK2309. The patients/participants provided their written informed consent to participate in this study.

## Author Contributions

EA designed the experiment, performed the measurements, discussed the data, and wrote the manuscript. WT designed the experiment, performed the measurements and analyzed the data, and contributed to the discussion of the manuscript. H-CJ discussed the data and contributed to writing of the manuscript. All authors contributed to the article and approved the submitted version.

## Conflict of Interest

The authors declare that the research was conducted in the absence of any commercial or financial relationships that could be construed as a potential conflict of interest.
